# Complete continuum of care for maternal health services and determinants among mothers who gave birth in the last year in Chiro City, Eastern Ethiopia: a community-based cross-sectional study (2024)

**DOI:** 10.3389/fgwh.2024.1404001

**Published:** 2024-09-06

**Authors:** Mohammed Ahmed Ali, Habtamu Geremew, Alegntaw Abate, Eyasu Bamlaku Golla, Mulat Belay Simegn, Hawi Kumbi, Smegnew Gichew Wondie, Samuel Abdisa, Temesgen Dessalegne Legasu, Moges Sisay Chekole

**Affiliations:** ^1^ Department of Midwifery, College of Health Sciences, Oda Bultum University, Chiro, Ethiopia; ^2^ College of Health Sciences, Oda Bultum University, Chiro, Ethiopia; ^3^ Department of Medical Laboratory Science, College of Health Sciences, Oda Bultum University, Chiro, Ethiopia; ^4^ Department of Public Health, College of Medicine and Health Sciences, Debre Markos University, Debre Markos, Ethiopia; ^5^ Department of Laboratory, Adama Hospital Medical College, Adama, Ethiopia; ^6^ Department of Human Nutrition, College of Medicine and Health Sciences, Mizan Tepi University, Mizan Aman, Ethiopia; ^7^ Department of Midwifery, Institute of Health Science, Jigjiga University, Jigjiga, Ethiopia; ^8^ Department of Midwifery, College of Medicine and Health Science, Debre Birhan University, Debre Birhan, Ethiopia

**Keywords:** continuum of care, maternal health, Chiro town, Ethiopia, Oda Bultum University

## Abstract

**Introduction:**

Ethiopia has one of the highest maternal death rates in the world, with 412 deaths for every 100,000 live births. A qualified healthcare professional's care during pregnancy, delivery, and the postnatal period is crucial to saving the lives of the mother and her child. The continuum of maternity health services in Ethiopia is still shallow, despite efforts to increase the use of these services. This study's goal was to evaluate the magnitude and factors influencing the entire continuum of care for maternal health services in Chiro City in 2024.

**Methods:**

A community-based cross-sectional study was conducted from 15 January to 15 February 2024, among mothers who gave birth in the last year preceding the data collection period in Chiro City, Eastern Ethiopia. Data were collected through face-to-face interviews using a structured questionnaire. Multivariable logistic regression analysis was done to identify determinants of the complete continuum of care. A *P*-value of <0.05 was considered statistically significant in this study.

**Result:**

The prevalence of a complete continuum of care among mothers who gave birth in the last year in Chiro District was 37.0%. Accordingly, women who received ANC were 5.24 times more likely to complete the maternal health services continuum of care compared with their counterparts (AOR: 5.24, 95% CI: 1.94, 14.15). Compared with their counterparts, women who received health education were 4.36 times more likely to complete the continuum of care (AOR: 4.36, 95% CI: 2.0, 9.51). Similarly, women who had planned pregnancies were 4.32 times more likely to complete the maternal health services continuum of care than those who had unplanned pregnancies (AOR: 4.32, 95% CI: 2.38, 7.85).

**Conclusion and recommendation:**

The continuum of care was favorably correlated with ANC usage, health education, and planned pregnancies. To increase the coverage of the entire continuum of maternal care, it is desirable to develop programs that encourage women to initiate ANC contacts, provide health education, and promote planned pregnancies.

## Introduction

Maternal mortality remains a serious health concern worldwide with an estimated 810 fatalities per day in 2017. The majority of these fatalities (94%) occurred in low-resource settings (1). The regions with the greatest rates of reported global maternal (86%) and newborn (80%) mortality are sub-Saharan Africa (SSA) and Southern Asia (2). Currently, Ethiopia has one of the highest MMRs in the world (412 fatalities per 100,000 live births). The main direct causes of maternal death include postpartum hemorrhage, hypertensive diseases of pregnancy, sepsis, and abortion (3). There is still an opportunity to prevent thousands of deaths among mothers and babies as these deaths are caused by complications related to pregnancy and childbirth (4).

The key intervention to save the mother's and her child's lives is the care provided by a skilled healthcare professional during pregnancy, labor, and the postnatal period (5). However, many women do not receive all of the services offered by the continuum of maternal healthcare, particularly in the country with the highest maternal mortality rates [antenatal care (ANC), skilled delivery care, and postnatal care (PNC)] (6).

A fundamental tenet of maternal, newborn, and child health (MNCH) programs aimed at reducing the number of maternal, neonatal, and pediatric fatalities is the continuum of care for maternal health (7). This continuum of care for maternal health services includes integrated service delivery for mothers and children from prepregnancy to delivery, the immediate postnatal period, and childhood. It is one of the important long-term plans for reducing maternal and neonatal deaths and improving the health and well-being of mothers and newborns (8).

It is a crucial long-term strategy for reducing the number of maternal and infant mortalities and enhancing the health and well-being of both groups of people. Offering suitable maternal health care services on a continuum can reduce avoidable causes of maternal and newborn mortality (9).

The place and time, or level of care, are two important elements that are highlighted by the continuum of maternity care. The temporal component underscores the significance of connections between the various packages of maternity care services that are offered during the course of pregnancy, childbirth, and the postpartum phase. The home, primary, secondary, and tertiary levels of care in healthcare delivery are all included in the place or level of care dimension (10).

The percentage of individual MCH care package utilization in Ethiopia improved between 2016 and 2019, according to data from the Ethiopian Demographic Health Survey (EDHS); the ANC went from 62% to 74%, the SBA from 28% to 50%, and the PNC from 17% to 34% (11).

Ethiopia's range of maternity health services is still shallow despite efforts to increase the use of these services; further research is necessary in this area (12). In Ethiopia, few women complete the recommended continuum of care, (11.2% in Legambo District, 16.1% in Jimma, 21.6% in the North West of Ethiopia, 21.5% in West Shoa, 37.2% in Debre Birhan and Benishangul Gumuz, 42.4% in Mekane Selam Town, and 45% in Enemay District) (1, 4, 9, 10, 13–15). Another study from rural areas of Ethiopia showed that there was a very high drop in maternal health service uptake along the continuum, with only one in seven women completing the continuum of care (16). In a multilevel analysis study conducted in Ethiopia, the variance in the odds of completing a continuum of care was attributed to the between-community level of only 24% (17). In other multi-analysis studies in Ethiopia, the overall prevalence of completion of the continuum of maternity care was only 12.9% (18). Therefore, determining the extent of the maternal continuum of health care and related factors is crucial for interventions aimed at reducing the continuum of care failure (19).

According to a study conducted in Northwestern Ethiopia, educational status, region of residence, and wealth index of the women were associated factors affecting the maternal continuum of care (12). In a prospective study conducted in Jimma, residency, women's occupation, parity, and the educational status of the partner were associated factors affecting the utilization of the continuum of care for maternal health services (13). In a recent study conducted in Mekane Selam Town, Amhara Region, prepregnancy contraceptive utilization, planned pregnancy, timing of first ANC contact, and educational status of the women were significant factors (14).

There is a dearth of knowledge about the prevalence and factors associated with mothers completing the continuum of maternity healthcare in the study area, West Hararghe Zone, Ethiopia, despite the fact that some attempts have been made thus far to address the status of maternal healthcare service utilization discretely. These shortcomings have led to an inadequate level of intervention throughout the spectrum of maternity care. Thus, the purpose of this study was to evaluate the scope and factors influencing the full continuum of care for maternal health services in the Chiro District in 2024.

## Methods and materials

### Study settings and period

A community-based cross-sectional study was conducted from 15 January to 15 February 2024, among mothers who gave birth in the last year preceding the data collection period in Chiro City, Eastern Ethiopia. It is one of Oromia National Regional State's 21 zones. This town has 33,670 residents overall as of the current national census, with 15,552 females and 18,118 males. The majority of people living there identified as Muslims, with 49.88% of them saying they adhered to this religion; 43.34% of them were followers of Ethiopian Orthodox Christianity, and 5.33% of them were Protestants (20).

### Populations

The source population consisted of all women who had given birth within the previous year and had resided in the district; the study population consisted of those who had been in Chiro for 6 months or more and had been living in the chosen kebeles during the data collection period. The study excluded women who were less than 42 days postpartum at the time of data collection as those mothers were not completing the maternal services given in the postpartum period.

### Sample size determination and sampling procedure

The calculation for the single population proportion was used to determine the sample size. The anticipated percentage of a full continuum of maternity care coverage is 16.1% based on a similar survey carried out in Jimma, Oromia Regional State (13), the margin of error is 5%, and the confidence interval is 95%. The required sample size was calculated as follows:

$$\frac{n=\;z{\left(\frac{\alpha }{2}\right)}^{2}\times p(1-p)}{d^{2}}$$

Where,

*N*: sample size

z $\frac{\alpha }{2}$: 95% confidence interval (1.96)

*d*: 5% margins of error

*P*: expected proportion of coverage of the continuum of maternal healthcare (16.1%)

$$n=\frac{{\left(1.96\right)}^{2}\times 0.42(1-0.42)}{{\left(0.05\right)}^{2}}=204$$

The minimum sample size needed with the aforementioned inputs is 204. The ultimate sample size is 336 when the non-response rate of 10% and the design effect of 1.5 are adjusted.

Initially, 4 kebeles, namely, Chafe Arara, Ifa Najabas, Alawagora, and Birka Chiro, from Chiro City were selected using the lottery method from a total of 10 kebeles, by using a simple random sampling technique (one-third of the total). Next, based on the number of mothers who gave birth in each kebele during the year prior to the data collection period (data gathered from the zonal health bureau), a proportionate allocation was implemented for each of the chosen kebeles.

### Data collection tools and procedures

Face-to-face interviews were used to gather data, and a structured questionnaire modified from an earlier study was used (9, 14, 15, 17, 21, 22). Four BSc nurses and four BSc midwifery professionals handled the data collection. The questionnaire was initially created in English and was translated into Oromiffa, the native tongue. Ultimately, to maintain consistency, we translated it back into English. Five percent of the samples in the Balina Chiro kebele, which was not part of the actual research regions, were pretested. Any inconsistency or inaccurate information was then adjusted appropriately. The principal investigator spent 2 days training supervisors and data collectors. Every day, the data were regularly checked for consistency and completeness. The principal investigator cleaned and entered the data.

### Measurement of variables

The outcome variable for maternal health services in this study was the full continuum of treatment. The sociodemographic variables were the mother's age, her educational background, her occupation, and her husband's educational background and occupation; the reproductive and obstetric variables include parity, the use of a prenatal contraceptive method, attending an ANC appointment for a recent pregnancy, the number of ANC contacts, knowledge of pregnancy danger signs, information about a birth attendant and postnatal care, the place of the birth, and the mother's postnatal care; and variables pertaining to personal and access to health care services (mothers’ autonomy in making decisions about their health care, education about maternal health care services, distance from the nearest health facility, and media exposure).

Maternal healthcare service utilization during pregnancy, delivery, and the postpartum period was measured to assess.

### Data processing and analysis

Prior to data analysis, data cleaning and cross-checking were completed. After being verified for accuracy and coded, the data were entered into EPI Data version 4.6 and exported to the Statistical Package for Social Science (SPSS) version 25.0 for further analysis. A logistic regression analysis, both multivariable and bivariable, was used to determine the relationship between the outcome and the explanatory variables.

Descriptive statistics including graphs, tables, and proportions were used to describe the data. Factors in bivariable analysis with a *P*-value of <0.2 were entered into the multivariable logistic regression model. Finally, multivariable logistic regression analysis was done to identify determinants of a complete continuum of care for maternal health services. Stepwise backward logistic regression was used to come up with the final model. A *P*-value of <0.05 was considered statistically significant in this study.

## Result

### Sociodemographic characteristics of the participants

In this study, a total of 335 participants were enrolled, yielding a response rate of 99.7%. The mean age of the study participants was 20.19 years (SD 3.7). Approximately half of the participants (47.8%) were Muslims. More than half of the respondents (68.1%) were married. The predominant ethnic group was Oromo (71.6%). In terms of their educational status, approximately one-third of the women (33.4%) and husbands (30.4%) had attained primary education (Table 1).

**Table 1:** Sociodemographic characteristics of study participants among mothers who give birth in the last 1 year in Chiro District, Eastern Ethiopia, 2024 (*n* = 335).

Variable	Category	Frequency	Percentage (%)
Age	15–25	69	20.6
	26–35	135	40.3
	36–49	131	39.1
Religion	Muslim	160	47.8
	Orthodox	109	32.5
	Protestant	41	12.2
	Other	25	7.5
Marital status	Single	28	8.4
	Married	228	68.1
	Divorced	45	13.4
	Widowed	34	10.1
Ethnicity	Oromo	240	71.6
	Amhara	47	14.0
	Somali	30	9.0
	Other	18	5.4
Maternal education	No formal education	90	26.9
	Primary education	112	33.4
	Secondary education	78	23.3
	College and above	55	16.4
Maternal occupation	Housewife	57	17.0
	Private employe	127	37.9
	Government employee	73	21.8
	Merchant	55	16.4
	Daily laborer	16	4.8
	Other	7	2.1
Husband education	No formal education	51	15.2
	Primary education	102	30.4
	Secondary education	94	28.1
	College and above	88	26.3
Husband occupation	Private employe	36	10.7
	Government employee	127	37.9
	Merchant	95	28.4
	Daily laborer	56	16.7
	Other	21	6.3

### Reproductive and obstetric variables of participants

In this study, regarding parity, half of the respondents (50.7%) had one or two children. More than half of the respondents (57.3%) did not use prepregnancy contraceptive methods, and 52.5% of the respondents reported that they received less than four ANC contacts. The majority of the participants (84.5%) received antenatal care during their recent pregnancy. Approximately 70.4% of the study participants had information regarding danger signs during pregnancy, and 46.3% of the respondents reported that they were not informed about birth preparedness and the complication readiness (COR) plan. Approximately 56.7% of the respondents had given birth in a health facility, while 53.7% of the women reported that they were assisted by health professionals during their recent delivery. Approximately 55.5% of the respondents received postnatal care within 24 h (Table 2).

**Table 2:** Reproductive and obstetric characteristics of study participants among mothers who give birth in the last 1 year in Chiro District, Eastern Ethiopia, 2024 (*n* = 335).

Variable	Category	Frequency	Percentage (%)
Parity	1–2	170	50.7
	3–4	90	26.9
	5 and above	75	22.4
Prepregnancy utilization of contraceptives	Yes	143	42.7
	No	192	57.3
Was recent pregnancy planned?	Yes	159	47.5
	No	176	52.5
Did you receive ANC?	Yes	283	84.5
	No	52	15.5
Number of ANC contacts	Less than 3 times	69	24.4
	4 and above	214	75.6
Know the danger signs of pregnancy	Yes	239	70.4
	No	99	29.6
Information on the BPRC plan	Yes	155	46.3
	No	180	53.7
Place of delivery	Health facility	190	56.7
	Home	145	43.3
Birth attendant	Health professional	180	53.7
	Traditional birth attendant	106	31.6
	Other	49	14.6
PNC service	Yes	218	65.1
	No	117	34.9
Time of PNC service	Within 24 h	187	85.77
	25–48 h	13	5.96
	49–72 h	10	4.58
	73 h–7 days	6	2.75
	8–42 days	2	0.91

### Personal and access to health service-related variables

A total of 78.2% of the study participants had autonomy in making health care decisions. Approximately 82.4% of the respondents received health education on maternal health care services. A total of 66.6% of the respondents had access to mass media (Table 3).

**Table 3:** Personal and access of health service-related characteristics of study participants among mothers who give birth in the last 1 year in Chiro District, Eastern Ethiopia, 2024, (*n* = 335).

Variable	Category	Frequency	Percentage (%)
Decision-making autonomy	Yes	262	78.2
	No	73	21.8
Health education	Yes	276	82.4
	No	59	17.6
Distance from district health facility	Less than 2 km	279	83.3
	2 km and above	56	16.7
Access to mass media	Yes	223	66.6
	No	112	33.4

### Magnitude of the complete continuum of care

The magnitude of the complete continuum of care for maternal health services among mothers who gave birth in the last year in Chiro District was 37.0% (CI: 31.82, 42.21). In this study, from the total participants, four and above ANC contacts were 63.9%, health professional birth attendants were 60.6%, and uptake of PNC services within 24 h was 90.4%.

### Factors associated with the complete continuum of care for maternal health services

The study employed both bivariate and multivariate logistic regression analysis to determine the factors linked to a full continuum of care for maternal health services. Eight variables from the bivariable logistic regression analysis were included in the final multivariable logistic regression model, with a significance level of less than 0.2. The variables included the autonomy of the mother in making decisions, the use of contraceptives prior to pregnancy, health education, the distance from the district health institution (less than 2 km), the use of ANC, planned pregnancy, information about the birth attendant (medical professionals), and the complication readiness plan. Three variables were shown to be statistically significant when paired with the continuum of maternal healthcare services in the multivariable logistic regression study.

Accordingly, women who received ANC were 5.24 times more likely to complete the maternal health services continuum of care than those who did not receive ANC [adjusted odd ratio (AOR): 5.24, 95% CI: 1.94, 14.15]. Compared with their counterparts, women who received health education were 4.36 times more likely to complete the maternal health services continuum of care (AOR: 4.36, 95% CI: 2.0, 9.51). Similarly, women who had planned pregnancies were 4.32 times more likely to complete the maternal health services continuum of care than women who had unplanned pregnancies (AOR: 4.32, 95% CI: 2.38, 7.85) (Table 4).

**Table 4:** Bivariable and multivariable logistic regression analysis of factors associated with a complete continuum of maternity care among mothers who gave birth in the last 1 year in Chiro District, Eastern Ethiopia, 2024 (*n* = 335).

Variable	Continuum of care		COR (95% CI)	AOR (95% CI)
	No	Yes		
Prepregnancy utilization of contraceptives				
Yes	56	87	1.43 (0.91, 2.25)	1.20 (0.68, 2.23)
No	68	124	1	1
Did you receive ANC?				
Yes	118	165	5.48 (2.26, 13.25)	5.24 (1.94, 14.15)*
No	6	46	1	1
Planned pregnancy				
Yes	38	143	1	1
No	86	68	4.75 (2.94, 7.68)	4.32 (2.38, 7.85)*
Information on the BPRC plan				
Yes	46	109	1.81 (1.15, 2.85)	0.55 (0.30, 0.99)
No	78	102	1	1
Birth attendant				
Health professional	105	73	3.87 (1.92, 7.80)	2.36 (1.09, 8.51)**
Traditional birth attendant	6	100	0.16 (0.05, 047)	0.14 (0.14, 0.43)
Other	13	36	1	
Decision-making autonomy				
Yes	101	161	1.36 (0.78, 2.34)	0.79 (0.35, 1.79)
No	23	50	1	1
Health education				
Yes	116	160	4.62 (2.11, 10.11)	2.50 (0.68, 2.23)
No	8	51	1	1
Distance from district health facility				
Less than 2 km	115	164	3.36 (1.72, 7.76)	0.40 (0.06, 2.53)
2 km and above	9	47	1	1

1, reference category.

* Significant at *p* < 0.05.

** Significant at *p* < 0.001.

## Discussion

This study examined the magnitude of the continuum of care for maternal healthcare services and its determinant factors among women who gave birth in the last year preceding this study. Overall, 37% of mothers completed the continuum of care for maternal healthcare services in the Chiro District.

The results of this study are in line with those of previous studies conducted in the Debre Birhan (37.2%), Mekane Selam (42.4%), and Enemay Districts (45%) (14, 10, 13). This similarity might be due to study design similarity (community-based cross-sectional study design) and comparable living conditions of the population.

The results of this study were greater than those of studies conducted in Nigeria (6.5%), Legambo District (11.2%), Jimma District (16.1%), Wayu District (16.1%), and Northwestern Ethiopia (21.6) (8, 12, 19). This discrepancy might be due to differences in sample size and sociodemographics. Hence, our study was conducted only in urban areas, while the compared studies were performed at a general level and specifically in rural areas, which provide a relatively greater chance of accessing maternal health services. This finding was supported by studies showing that being a rural resident negatively affects the likelihood of receiving a full continuum of maternal care compared with being a rural resident.

The results of this study are lower than those of studies conducted in Egypt (50.4%) and Cambodia (60%) (20, 21). This difference might be due to variations in geographic location, access to health and infrastructure, healthcare coverage, and the accessibility of services. Hence, the inclusion of those who had at least one ANC contact and who had PNC within 48 h or 6 weeks may have contributed to the variance compared to the current study. The other possible explanation could be that a retrospective study period longer than five years prior to the survey might increase the participants’ recall bias about the services they received for the last 5 years.

In this study, women who received ANC were five times more likely to complete the continuum of health services compared with their counterparts. This finding is in line with the study conducted in Debre Birhan (11). This might be explained by women who did not receive ANC having a poor birth preparedness plan, lower knowledge of the expected date of delivery, and a lower likelihood of having a birth plan in terms of the desired place of delivery and preferred birth attendant.

In this study, women who received health education were 2.36 times more likely to complete the continuum of health services compared with their counterparts. This finding is in line with other studies (1, 9). This might be because women are better equipped to handle emergencies and are aware of the danger signs of pregnancy and the postpartum period. An excellent opportunity to become comfortable with the surroundings of the medical facility is provided by providing health education, which would have helped them avoid the unwarranted anxiety and tension associated with using maternal health services. In addition, health education about birth preparedness encouraged women to complete the full continuum of maternity care more frequently.

In this study, women who had planned pregnancy were 4.32 times more likely to complete the continuum of health services compared with their counterparts. This finding is in agreement with other studies (4, 9, 19, 22). The reason could be because, in comparison to women who are pregnant against their will, those who are pregnant because they want to be pregnant are more likely to be cautious about their health, become more motivated, and be emotionally and financially ready for the demands of pregnancy and childbearing.

## Conclusion and recommendations

In general, this study employed an integrated assessment of the full continuum of care from antenatal to postpartum stages. The findings indicated that only 37% of women in the district received the full continuum of care, which is less than the recommended coverage by the Ministry of Health and the World Health Organization and falls short of expectations. In this study, the full continuum of care was favorably correlated with ANC usage, planned pregnancy, and providing health education. To increase the coverage of the entire continuum of maternal care, it is therefore desirable to develop programs that encourage women to initiate ANC contacts, promote planned pregnancies, and provide health education.

### Strengths and limitations of the study

This finding is considered to be the first to identify the prevalence and determinants of the continuum of care for maternal health services in the study area. Because the women in the study were asked to recollect their pregnancies and deliveries, recall bias may have occurred as these women were in the postpartum period. We kept the source population within a year of giving birth in an effort to minimize recall bias.

## Data Availability

The original contributions presented in the study are included in the article/Supplementary Material, further inquiries can be directed to the corresponding author.

## References

[1] Atnafu A Kebede A Misganaw B Teshome DF Biks GA Demissie GD Determinants of the Continuum of maternal healthcare services in northwest Ethiopia: findings from the Primary Health Care Project. J Pregnancy. (2020) 2020:4318197. 10.1155/2020/4318197 32908704 PMC7471826

[2] Health C Analysis M. Factors associated with the completion of the continuum of care for maternal, factors associated with the completion of the continuum of care for maternal, newborn, and child health services in Ethiopia. Multilevel Model Analysis. (2021) 26:1.

[3] Hussen AM Ibrahim IM Tilahun B Tunçalp Ö Grobbee DE Browne JL. Completion of the Continuum of maternity care in the emerging regions of Ethiopia: analysis of the 2019 Demographic and Health Survey. Int J Environ Res Public Health. (2023) 20:6320. 10.3390/ijerph20136320 37444167 PMC10342030

[4] Shitie A Assefa N Dhressa M Dilnessa T. Completion and factors associated with maternity continuum of care among mothers who gave birth in the last one year in Enemay District, Northwest Ethiopia. J Pregnancy. (2020) 2020:7019676. 10.1155/2020/7019676 32953177 PMC7481927

[5] Sewunet T Id A Teshale AB Yeshaw Y Alem Z Ayalew HG Barriers for health care access affects maternal continuum of care utilization in Ethiopia; spatial analysis and generalized estimating equation. PLoS One. (2022) 17:1–18. 10.1371/journal.pone.0266490 PMC9032438 35452475

[6] Tesfay N Hailu G Woldeyohannes F. Effect of optimal antenatal care on maternal and perinatal health. Matern Perinat Heal. (2023) 11:1–12. 10.3389/fped.2023.1120979 PMC9941639 36824654

[7] James KS Mishra US Rinju Pallikadavath S. Sequential impact of components of maternal and child health care services on the continuum of care in India. J Biosoc Sci. (2022) 54:450–72. 10.1017/S002193202100016X

[8] Surveys H. Completing the Continuum of Care for Maternal and Newborn Health in Cambodia: Who Drops out? Calverton: ICF International Calverton (2013).

[9] Cherie N Abdulkerim M Abegaz Z. Maternity continuum of care and its determinants among mothers who gave birth in Legambo District, South Wollo, Northeast Ethiopia. Health Sci Rep. (2021) 4:2. 10.1002/hsr2.409 PMC8562404 34754945

[10] Buli TD Wakgari N. Completion of the continuum of maternity care and associated factors among women who gave birth in the last months in Chelia District, West Shoa Zone, Ethiopia: a community-based cross-sectional study. Front Public Health. (2023) 10. 10.3389/fpubh.2022.1026236 36684973 PMC9845613

[11] Tizazu MA Sharew NT Mamo T Zeru AB Assefa EY Amare NS. Completing the Continuum of maternity care completing the continuum of maternity care and associated factors in Debre Berhan Town, Amhara, Ethiopia, 2020. J Multidiscip Healthc. (2022) 32:20–32. 10.2147/JMDH.S293323 PMC7797327 33442260

[12] Tsega D Admas M Talie A Tsega TB Birhanu MY Alemu S Maternity continuum care completion and its associated factors in Northwest Ethiopia. J Pregnancy. (2022) 2022:6–7. 10.1155/2022/1309881 PMC8872697 35223099

[13] Kitila SB Feyissa GT Wordofa MA. Completion of the maternal health care continuum — barriers and facilitators among pregnant women in Jimma Zone, Southwest Ethiopia: a prospective study. Health Serv Insights. (2023) 16:4–6. 10.1177/11786329231214607 PMC10691321 38046557

[14] Mengistie HT Belay MA Sendekie AD Shitie A Sewyew DA. Complete continuum of maternity care and associated factors among mothers who gave birth in the last twelve months in Mekane Selam Town North-East Ethiopia. PLoS One. (2023) 18(9):1–15. 10.1371/journal.pone.0289200 PMC10538653 37768954

[15] Zelka MA Yalew AW Debelaw GT. Completion and determinants of a continuum of care in maternal health services in Benishangul Gumuz Region: a prospective follow-up study. Front Public Health. (2022) 4:5–7. 10.3389/fpubh.2022.1014304 PMC9672503 36407997

[16] Dadi TL Medhin G Kasaye HK Kassie GM Jebena MG Gobezie WA Continuum of maternity care among rural women in Ethiopia: does place and frequency of antenatal care visit matter? BMC Reprod Health. (2021) 18:1–12. 10.1186/s12978-021-01265-x PMC8572478 34742315

[17] Emiru AA Alene GD Debelew GT. Women’s retention on the continuum of maternal care pathway in West Gojjam Zone, Ethiopia: multilevel analysis. BMC Pregnancy Childbirth. (2020) 20:258. 10.1186/s12884-020-02953-5 32349687 PMC7191802

[18] Abebe GF Belachew DZ Girma D Aydiko A Negesse Y. Multilevel analysis of the predictors of completion of the continuum of maternity care in Ethiopia; using the recent 2019 Ethiopia mini demographic and health survey. BMC Pregnancy Childbirth. (2022) 22:1–13. 10.1186/s12884-022-05016-z 36071407 PMC9450293

[19] Tadese M Tessema SD Aklilu D Wake GE Mulu GB. Dropout from a maternal and newborn continuum of care after antenatal care booking and its associated factors in Debre Berhan Town, Northeast Ethiopia. Front Med. (2022) 9:4–7. 10.3389/fmed.2022.950901 PMC9558816 36250090

[20] From Wikipedia the free encyclopedia. Chiro (town). (2021).

[21] Sertsewold SG Debie A Geberu DM. Continuum of maternal healthcare services utilisation and associated factors among women who gave birth in Siyadebirena Wayu District, Ethiopia: community- ­ based cross- ­ sectional study. BMJ Open. (2021) 7:1–15. 10.1136/bmjopen-2021-051148 PMC8627416 34824117

[22] Hamed AF Roshdy E Sabry M. Egyptian status of continuum of care for maternal, newborn, and child health: Sohag Governorate as an example. Int J Med Sci Public Health. (2018) 7(6):417–26. 10.5455/ijmsph.2018.0102607032018

